# Wage Earners and Sickness

**Published:** 1903-01-03

**Authors:** 


// The Hospital.

A Journal of

The Medical Sciences and Hospital Administration.

Vol. XXXIII.—No. 849.] Edited by Sir Henry Burdett, K.C.B., and Solomon C. Smith, M.D., M.R.C.P. [January 3, 1903.

### Wage Earners and Sickness.

The letter from Dr. Heron, on the Industrial Assurance Societies of Germany, which appeared in the *Times* of December 26th, is a document calling for the careful consideration of all who are interested in the provision of medical attendance for the wage-earning classes of the community. The questions hence arising have, as is only too well known, been productive from time to time of considerable friction, not only between individual medical men and the benefit or sick societies to which they were attached, but also between committees more or less representative of the profession as a whole, and delegates or other persons appointed by the managers of trade-unions or kindred bodies. Dr. Glover, during his membership of the General Medical Council, was honourably distinguished, by his efforts to bring about an understanding between the administrators and the recipients of medical assistance, and to establish, by negotiations with the heads of the great friendly societies of this country, a better understanding than now exists with regard to the basis on which medical attendance upon large bodies of men and their families might be so arranged as to be efficacious as regards the sick, and reasonably profitable to the practitioners who were concerned in conducting it. The Medical Council, as such, had obviously no concern with the affair; but its members good naturedly permitted the ægis of its name to be thrown over the proceedings of a committee specially appointed to confer with the representatives of the friendly societies; and it was no doubt hoped that the "Council" would represent, to the minds of these representatives, an authority as dim and vast as is that of the "Lormaire" to the average Frenchman. The hope, if it existed, was doomed to disappointment; for the "Societies" were not sufficiently enlightened to perceive that services obtained for three-halfpence were not likely to be worth any materially larger sum. The general result has been that the wage-earning classes have continued, perhaps with a few exceptions, to buy their medical advice in the cheapest market, and that medical men who believe in the efficacy of invocations of Hercules have from time to time appealed to the Medical Council to declare that certain forms of contract were professionally "infamous" in their character. To such appeals the Council, not having finally said good-bye to common sense, has been compelled to turn a deaf ear, and matters generally have remained very much as they were when the questions at issue were first agitated. At one great industrial centre, indeed, they have been rendered worse by a foolish endeavour to set up a so-called "consulting" institution at a cheap rate, an endeavour which, in addition to being foolish, unfortunately served to afford an illustration of the possible folly of the generally wise.

While such is the state of things in Great Britain, and while the somewhat related question of old-age pensions for wage-earners appears to be still a long way from practical realisation, it would appear from Dr. Heron's letter that both this and the care of the sick have been accomplished in Germany, not by the intervention of politicians or philanthropists, but by the thrift and common sense of the working classes themselves, aided by legislation adapted to their needs. The German workman has determined to banish the curse of squalid poverty from his class; and his object has been effected by an insurance fund to which employers and workers alike are compelled to contribute, and which is further assisted by a grant from the State. The capital of this fund now amounts to more than sixty millions of pounds sterling, and is to be materially increased; while those to whom its management is committed have realised the fact that nothing is so costly to the individual or the community as disabling illness, and that nothing is so economical as to incur any necessary expense in order that illness may be cured, or, if not curable, that it may be confined within the narrowest attainable limits. To this end hospitals or sanatoria have been erected in various parts of the country, not by almsgivers or by so-called "charity," but by money belonging to the classes who will benefit by them; and to these institutions the wage-earner has a right of admission, by virtue of his contributions to the general fund, whenever his state is such as to render such admission desirable. Dr. Heron describes the hospital at Beelitz as an almost palatial establishment, fitted with every appliance which can be conducive either to the advancement of science or to the recovery of the inmates; and it seems certain that what is possible in Germany would be equally possible in England; but it is also certain that the machinery which has been successful in the former country would not be likely to prove equally effective in the latter. Whatever we do, we must do in our own way; and, even if aiming at the same result, we must be content to employ different means. Would it not be possible, in some great manufacturing town, for a medical man of influence and position to take the initiative in a scheme for the provision of a self-supported hospital, and to enlist the wage-earners of the locality in its cause. For a time, no doubt, they would be some opposition, but truth would in the end be likely to prevail, and the men who carried out such a work as that which we have suggested would be certain to attain permanent positions of public and political influence.

